# Increased estrogen to androgen ratio enhances immunoglobulin levels and impairs B cell function in male mice

**DOI:** 10.1038/s41598-020-75059-9

**Published:** 2020-10-27

**Authors:** Juan Antonio Aguilar-Pimentel, Yi-Li Cho, Raffaele Gerlini, Julia Calzada-Wack, Maria Wimmer, Philipp Mayer-Kuckuk, Thure Adler, Carsten B. Schmidt-Weber, Dirk H. Busch, Helmut Fuchs, Valérie Gailus-Durner, Markus Ollert, Martin Hrabě de Angelis, Claes Ohlsson, Matti Poutanen, Raffaele Teperino, Leena Strauss

**Affiliations:** 1 German Mouse Clinic, Institute of Experimental Genetics, Helmholtz Center Munich, German Research Center for Environmental Health, Ingolstädter Landstrasse 1, 85764 Neuherberg, Germany; 2 Institute of Experimental Genetics, Helmholtz Zentrum München, German Research Center for Environmental Health, Ingolstädter Landstrasse 1, 85764 Neuherberg, Germany; 3 German Center for Diabetes Research (DZD), Ingolstädter Landstrasse. 1, 85764 Neuherberg, Germany; 4 Institute of Environmental Medicine, UNIKA-T, Augsburg, Germany; 5 Centre of Allergy and Environment (ZAUM), IAF, Technische Universität Und Helmholtz Zentrum München, Munich, Germany; 6 Institute for Medical Microbiology, Immunology and Hygiene, Technische Universität München, Trogerstrasse 30, 81675 Munich, Germany; 7 Institute of Stem Cell Research, Helmholtz Zentrum München, German Research Center for Environmental Health, Ingolstädter Landstraße 1, 85764 Neuherberg, Germany; 8 Department of Infection and Immunity, Luxembourg Institute of Health, Esch-sur-Alzette, Luxembourg; 9 Department of Dermatology and Allergy Center, Odense Research Center for Anaphylaxis, University of Southern Denmark, Odense, Denmark; 10 Chair of Experimental Genetics, School of Life Science Weihenstephan, Technische Universität München, Alte Akademie 8, 85354 Freising, Germany; 11 Institute of Medicine, Sahlgrenska Academy, University of Gothenburg, Gothenburg, Sweden; 12 Institute of Biomedicine, University of Turku, Turku, Finland; 13 Turku Center for Disease Modeling, University of Turku, Turku, Finland

**Keywords:** Immunogenetics, Endocrine system and metabolic diseases

## Abstract

Sex steroids, such as estrogens and androgens, are important regulators of the humoral immune response. Studies in female mice have demonstrated that alteration of circulating estrogen concentration regulates antibody-mediated immunity. As males have normally little endogenous estrogen, we hypothesized that in males high estrogens and low androgens affect the immune system and enhance the allergic inflammatory response. Here, we studied transgenic male mice expressing human aromatase (AROM+). These animals have a high circulating estrogen to androgen ratio (E/A), causing female traits such as gynecomastia. We found that AROM+ male mice had significantly higher plasma immunoglobulin levels, particularly IgE. Flow cytometry analyses of splenocytes revealed changes in mature/immature B cell ratio together with a transcriptional upregulation of the Igh locus. Furthermore, higher proliferation rate and increased IgE synthesis after IgE class-switching was found. Subsequently, we utilized an ovalbumin airway challenge model to test the allergic response in AROM+ male mice. In line with above observations, an increase in IgE levels was measured, albeit no impact on immune cell infiltration into the lungs was detected. Together, our findings suggest that high circulating E/A in males significantly alters B cell function without any significant enhancement in allergic inflammation.

## Introduction

Steroid hormones including estradiol, progesterone and testosterone regulate the humoral immune response^1,2^. For example, estrogens have been shown to block the elimination of autoreactive B cells, and to induce production of autoantibodies^3^, whereas androgens have shown to inhibit the proliferation of potentially autoreactive B cells in the periphery^4^. Furthermore, estrogen-like compounds, such as equol, have shown to increase the production of allergen-specific IgE from ovalbumin-immunized BALB/c mice^5^ and to promote degranulation of mast cells in culture^6^. Together, these data demonstrated that the ratio of estrogen to androgen determines the B cell response. These important findings, notwithstanding, have been derived mostly from studies in female mice with altered estrogen levels. Furthermore, clinical data suggest a sexual bias at least in pathological immune conditions^7–10^. Thus, we hypothesized that a shifted E/A profoundly affect the immune system and enhance allergic inflammatory response in males.

To assess the effect of altered estrogen to androgen levels on immune system in males in vivo, we selected the established AROM+ mouse model^11,12^. In this model, human aromatase is ubiquitously expressed, resulting in elevated endogenous levels of estradiol and reduced level of testosterone, and hence, estrogen to androgen ratio (E/A) in male mice, yet estrogen levels remain physiological, i.e. similar to female mice. The results from our experiments demonstrated that increased circulating E/A in male mice leads to altered humoral immunity. In addition, we observed a shifted distribution of B cell subpopulation, an increased B cell proliferation rate in vitro, and changes in the gene expression in different the splenic B cell subpopulations.

## Results

### Sex steroids affect the production of immunoglobulins in vivo in males

**Figure 1:**
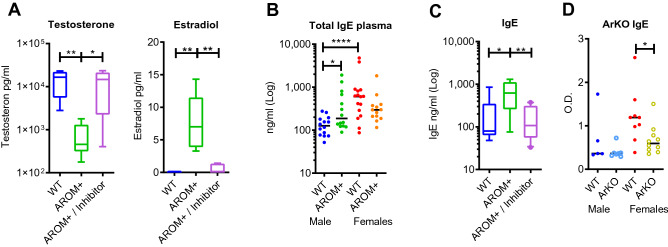
Aromatase inhibitor treatment normalizes the sex steroid levels and consequently IgE and IgG1 levels in AROM+ males. (**A**) Estradiol and testosterone concentrations at the age of 10 weeks (p70) in WT and AROM+ male mice with and without aromatase inhibitor treatment (n = 10 per group). (**B**) Total plasma IgE concentration in 7-week old (p48) AROM+ and WT males and females. There was a clear sex difference in WT animals, and the AROM+ males differed significantly from the WT male but not from the WT females (n = 13–16 per group). (**C**) Plasma IgE levels at the age of 10 weeks (p70) in WT and AROM+ male mice with and without aromatase inhibitor treatment: Inhibitor treatment rescued the increased IgE levels in AROM+ males (n = 10 per group). (**D**) Plasma IgE levels in the WT and ArKO male and female mice (n = 5–10 per group). The lack of functional aromatase enzyme significantly decreased the IgE levels in female mice. Data presented as median and interquartile range, Mann–Whitney test P: ≤ 0.05 (*), ≤ 0.01 (**), and ≤ 0.0001 (****).

To validate the AROM+ model for the study of E/A, levels of circulating estrogens and androgens were measured by gas chromatography tandem mass spectrometry. As previously shown^13^, male AROM+ mice have significantly higher estradiol and lower testosterone levels compared to wild type (WT) male mice. Furthermore, pharmacological inhibition of the aromatase activity resulted in the anticipated normalization of the hormone levels (Fig. 1A). Thus, the male AROM+ mice were confirmed to be suitable for studying E/A on the humoral immune response. Next, plasma immunoglobulin levels were measured, evidencing higher immunoglobulin IgE level in male AROM+ mice compared to WT male mice, measured at late pubertal age (48 days old, Fig. 1B). The levels were equivalent to WT females. Conformation that increased immunoglobulin levels are a direct consequence of an increased E/A in AROM+ males was obtained from pharmacological inhibition of the aromatase activity resulting in the anticipated normalization of the IgE levels (Fig. 1C). Additionally, animals lacking the functional aromatase enzyme, and consequently estrogen production (ArKO), had significantly lower IgE levels than their WT littermates (Fig. 1D).

**Figure 2:**
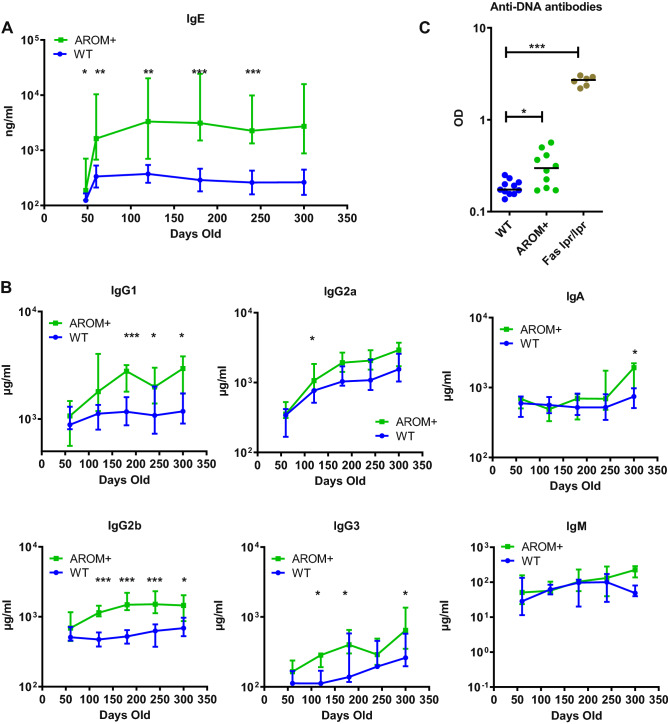
Increase in the estrogen to androgen ratio in AROM+ males enhances immunoglobulin production over time. (**A**) Differences in IgE levels between AROM+ and WT males was evident also during ageing (p48–p300, n = 5–14 per group). (**B**) The plasma levels of IgG1, IgG2a, IgA, IgG2b, IgG3 and IgM in AROM+ and WT males at different age (p60–p300). (**C**) The levels of anti-DNA antibodies in the plasma of the AROM+ males was significantly higher than in the WT males (p135, n = 6–11 mice per group), but lower than those measured in Fas lpr/lpr mice, those being a well-known autoimmune disease model. Data presented as median and interquartile range, Mann–Whitney test P: ≤ 0.05 (*), ≤ 0.01 (**) and ≤ 0.001 (***).

As immunoglobulin levels are largely age-dependent, we also measured their concentrations at different time points until late adult age (Fig. 2A). IgE levels in AROM+ males further increased after puberty and stayed significantly higher than in WT mice during the entire adult life (p60–p300). In addition to IgE, the concentrations of IgG1, IgG2a, IgG2b, IgG3 and IgA, but not IgM, were found significantly increased in AROM+ males compared to the age-matched WT males (Fig. 2B). Moreover, we found a significant increase in anti-DNA antibodies in the AROM+ plasma at the adult age (p135), although not being comparable to Fas lpr/lpr mouse, a well-known autoimmune disease mouse model (Fig. 2C). Thus, the increased levels of plasma immunoglobulin isotypes and anti-DNA antibodies levels are associated with allergy, autoimmune disorders or inflammatory diseases.

### Ovalbumin (OVA) airway inflammation challenge further increased the antibody response in AROM+ male mice

**Figure 3:**
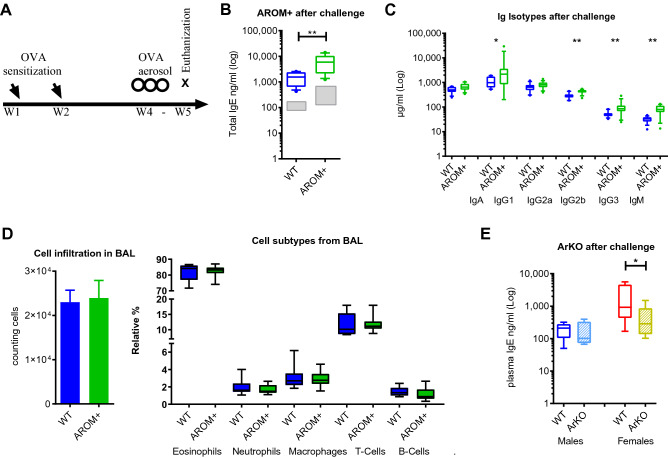
Increase in the estrogen to androgen ratio in AROM+ males enhanced the immunoglobulin response but did not interfere with the leukocyte recruitment in an acute allergic airway inflammation model. (**A**) Schematic presentation of the acute allergic airway inflammation model. The study weeks (W) for the different stimulations after initiation of the experiment is indicated. (**B**) Plasma concentration of IgE in the AROM+ and WT males after the challenge. Gray boxes indicate levels before the challenge. (**C**) Plasma IgG1, IGg2b, IgG3 and IgM levels in the AROM+ and WT males after the challenge. (**D**) Total infiltration of immune cells in the bronchial alveolar lavage (BAL) and the cell composition in the AROM+ and WT male mice after ovalbumin sensitization and challenging (mean ± SEM). (**E**) Plasma concentration of IgE in the male and female mice lacking aromatase enzyme (ArKO) after the challenge. n = 9–15 per group ~ 12 weeks). Data presented as median and interquartile range, Mann–Whitney test P: ≤ 0.05 (*), and ≤ 0.01 (**).

Based on the finding of significantly elevated baseline IgE levels in male AROM+ mice, the role of sex steroids in the regulation of allergy in vivo was further investigated in an established model of acute allergic airway inflammation (Fig. 3A). After sensitizing and challenging the mice to an allergen (ovalbumin), we found significantly increased amounts of IgE (Fig. 3B) and several other immunoglobulins (IgG1, IgG2b, IgG3 and IgM) in plasma samples of AROM+ males compared to WT (Fig. 3C). However, we could not detect differences in the amount or relative distributions of infiltrating leukocytes, such as eosinophils, macrophages, neutrophils, T or B lymphocytes in the bronchial alveolar lavage (BAL) between WT and AROM+ males mice (Fig. 3D). These findings suggest that an increased E/A enhances the production of IgE and other isotypes under steady-state and challenged conditions in male mice, but is not sufficient to impact on other immunological parameters such as the composition of the immune cells infiltrating in the bronchoalveolar space under an acute allergic airway inflammation model. Additionally, under the same experiment ArKO female mice show lower IgE levels than in WT females, both before and after allergen sensitization and challenge (Fig. 3E), together with no difference in cellular infiltration in BAL (data not shown). This indicates that IgE levels are reduced by the lack of estrogen but the inflammatory response is not affected. Interestingly, deficiency of estrogens in ArKO male mice neither affects the basal IgE concentration nor interferes with the ability of boosting IgE under airway inflammation challenge, indicating that estrogens are not essential for antibody production in male mice (Fig. 3E).

### Blood and spleen leukocyte populations in AROM+ males are more comparable to wild type females than males

**Figure 4:**
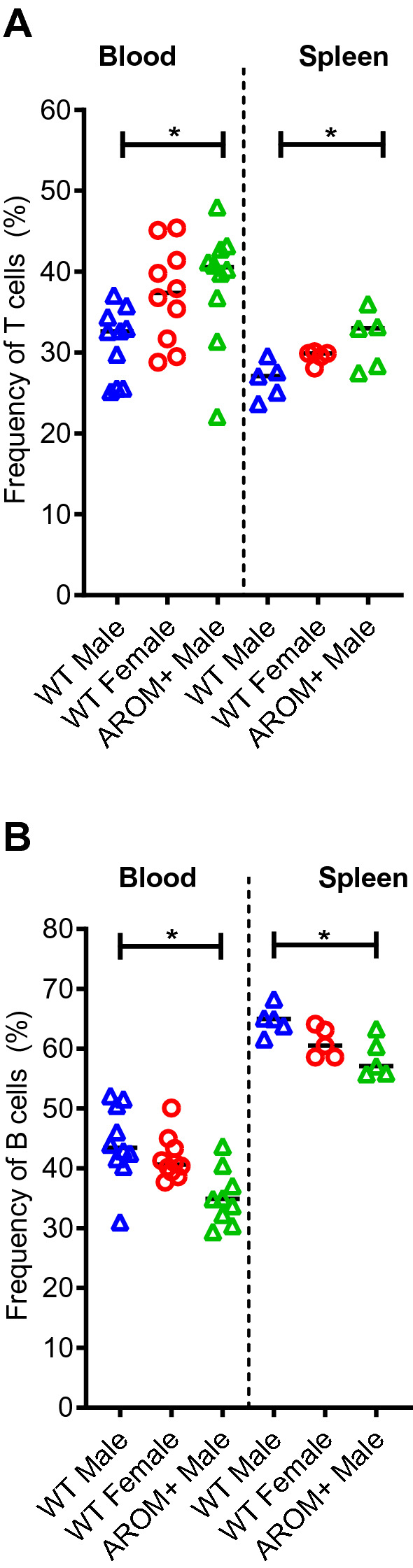
Flow cytometry analysis of the spleen and peripheral blood lymphocytes. Flow cytometry analysis indicated that the AROM+ males have higher T cell and lower B cell frequency than the wild type (WT) males. (**A**) The frequency of T cells in the AROM+, WT male, and WT female mice. (**B**) The frequency of B cells in the AROM+, WT male, and WT female mice. (n = 5–10 per group), (~ 44 weeks old) Data presented as median and scatter dot plot, Mann–Whitney test P: ≤ 0.05 (*).

The composition of T and B lymphocytes in AROM+ males in the whole blood and spleen was shifted to a wild type female pattern, i.e. the frequency of T cells was higher and the frequency of B cells lower in AROM+ males compared to WT males. The changes between WT and AROM+ males were even more dramatic than between genders, and therefore, cannot be simply explained by feminization (Fig. 4). Similarly, tendencies for sex-bias in the amounts of natural killer (NK) cells and eosinophils in blood were found, and AROM+ males resembled more the WT females than the WT males (Supplementary Fig. 1). Sex-bias in NK cells and eospinophils has been previously observed also in humans^14,15^ and in the splenic T and B cell frequences in mice with C57BL background in our large scale phenotyping screening of mice at the German Mouse Clinic (GMC) (Supplementary Fig. 2A). Furthermore, sex-based immune cell differences in other cells subtypes, such as pDCs, has been earlier described in humans^16^.

### The spleen B cells respond to altered circulating sex steroid levels

**Figure 5:**
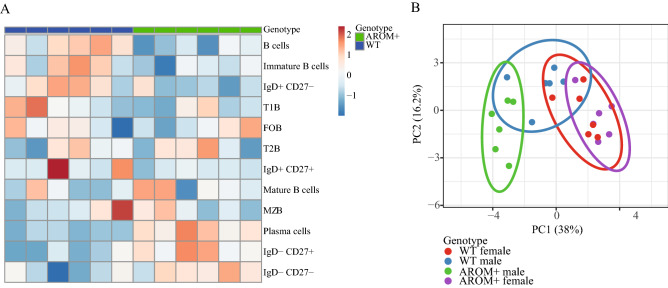
Flow cytometry analysis of the splenic B cells. (**A**) Heatmap from 6 WT and 6 AROM+ male mice showing differential expression of B cell markers in the spleen. The results suggest higher proportion of plasma cells (CD19^+^CD138^+^IgD^−^), switched memory cells (CD19^+^IgD^−^CD27^−^) and double negative switched memory cells (CD19^+^IgD^−^CD27^−^) in AROM+ males. (**B**) Principal Component Analysis of the gatings from female and male WT and AROM+ mice (n = 6 per group). The clustering shows a clear divergence of AROM+ male mice from the other groups.

As the immunoglobulin levels were altered in the male AROM+ plasma, a detailed flow cytometric analysis of the splenic B cells was performed. The analysis revealed an alteration of the composition of B cell subsets in the AROM+ male mice as compared with WT mice (Fig. 5A). In particular, we found an increased proportion of mature B cells (CD19^+^IgD^low^IgM^high^) along with a decrease in immature B cell fraction (CD19^+^IgD^high^IgM^low^). Moreover, the proportion of plasma cells (CD19^+^CD138^+^IgD^−^), switched memory cells (CD19^+^IgD^−^CD27^−^) and double negative (DN) switched memory cells (CD19^+^IgD^−^CD27^−^) was relatively higher in AROM+ male mice, which may contribute to the production of immunoglobulin IgE.

To visualize the cell-surface antigen expression, we applied principal component analyses (PCA)^17^ to our data (Fig. 5B). Interestingly, the PCA showed a clear independent cluster for AROM+ male, separate from AROM+ females and WT males and females, while the AROM+ and WT female mice clustered close together. The data indicate that the effect of an imbalanced estrogen to androgen ratio in spleen cells of male mice goes beyond a female phenotype pattern of the B cell fraction.

**Figure 6:**
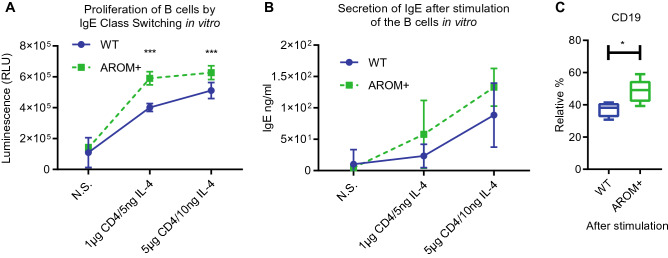
IgE class switching was significantly enhanced in the splenocytes isolated from AROM+ males compared to cells from WT males. (**A**) Proliferation of B cells after IgE class switching (mean ± SD, n = 6), (**B**) The secretion of IgE in B cells after class switching (mean ± SD, n = 6). (**C**) The relative amount of CD19 positive B cells in the AROM+ and WT splenocytes after the class switching. N.S.: no stimulation, data presented as median and interquartile range, Mann–Whitney test P: ≤ 0.05 (*), and ≤ 0.001 (***).

To evaluate the functionality of the splenic B cells in AROM+ male mice, we performed IgE immunoglobulin class switching in vitro by treating the cells with anti-CD40 and IL-4. In these studies, we found that AROM+ splenocytes have higher cell proliferation rate (Fig. 6A) and higher IgE synthesis than splenocytes from male controls after the class switching (Fig. 6B). Furthermore, there was a significant increase in CD45+ CD19+ B cells upon stimulation (Fig. 6C). These results indicated that splenic B cells from sex hormone-imbalanced (E/A) AROM+ male mice show enhanced proliferation and IgE class switching upon B cell activation.

**Figure 7:**
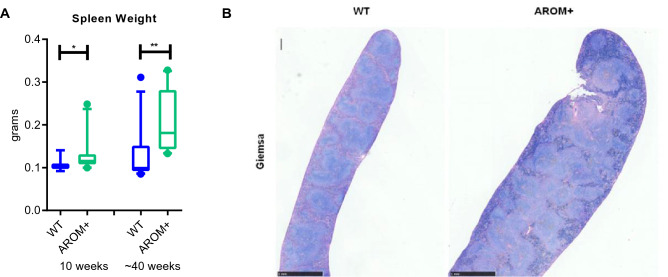
The enlargement of the AROM+ spleen. (**A**) The spleen weight of the AROM+ mice was increased already at young animals (10 weeks old), and the increase was even more pronounced at older age (40 weeks, n = 9–12 per group). (**B**) Representative micrographs of longitudinal sections of the spleen (Giemsa staining) at low magnification showing the architecture of the spleen. In the control mice, the distinction between the red and white pulp is clear. The AROM+ mice show diffuse follicular hyperplasia characterized by expansion and partial bridging of most of the areas of white pulp. Data presented as median and interquartile range, Mann–Whitney test P: ≤ 0.05 (*), and ≤ 0.01 (**).

Furthermore, we observed an enlargement of the spleen in AROM+ mice compared to the age-matched WT mice. While the increased weight of the spleen was observed already in young adults (10 weeks), the increase was even more evident at the age of 40 weeks (Fig. 7A). Interestingly, this is in line with our large scale phenotyping in the GMC which indicated that female mice have higher spleen weight than males in the C57BL genetic background (Supplementary Fig. 2B). The histological analysis revealed that the enlargement of the spleen was due to a diffuse follicular hyperplasia of the white pulp. The basic microanatomy of the white pulp periarteriolar sheath and marginal zone was maintained, but the follicles were enlarged. However, the relative presence of cells composed of macrophages, plasma cells, and T and B lymphocytes in the white pulp was normal (Fig. 7B and Supplementary Fig. 3). To find out, if the hormonal imbalance affect also the proportions of B, T_reg_ (regulatory T cells) or T_FH_ (follicular helper T cells) cells, we analyzed the compositions of the lymphocytes in the spleen, small intestine and Payer’s Patches. However, we could not find any major changes in their proportions under in vivo imbalanced estrogen to androgen ratio (E/A) (Supplementary Fig. 4).

### Estrogen and androgens regulated gene expression in splenic B cells of males

**Figure 8:**
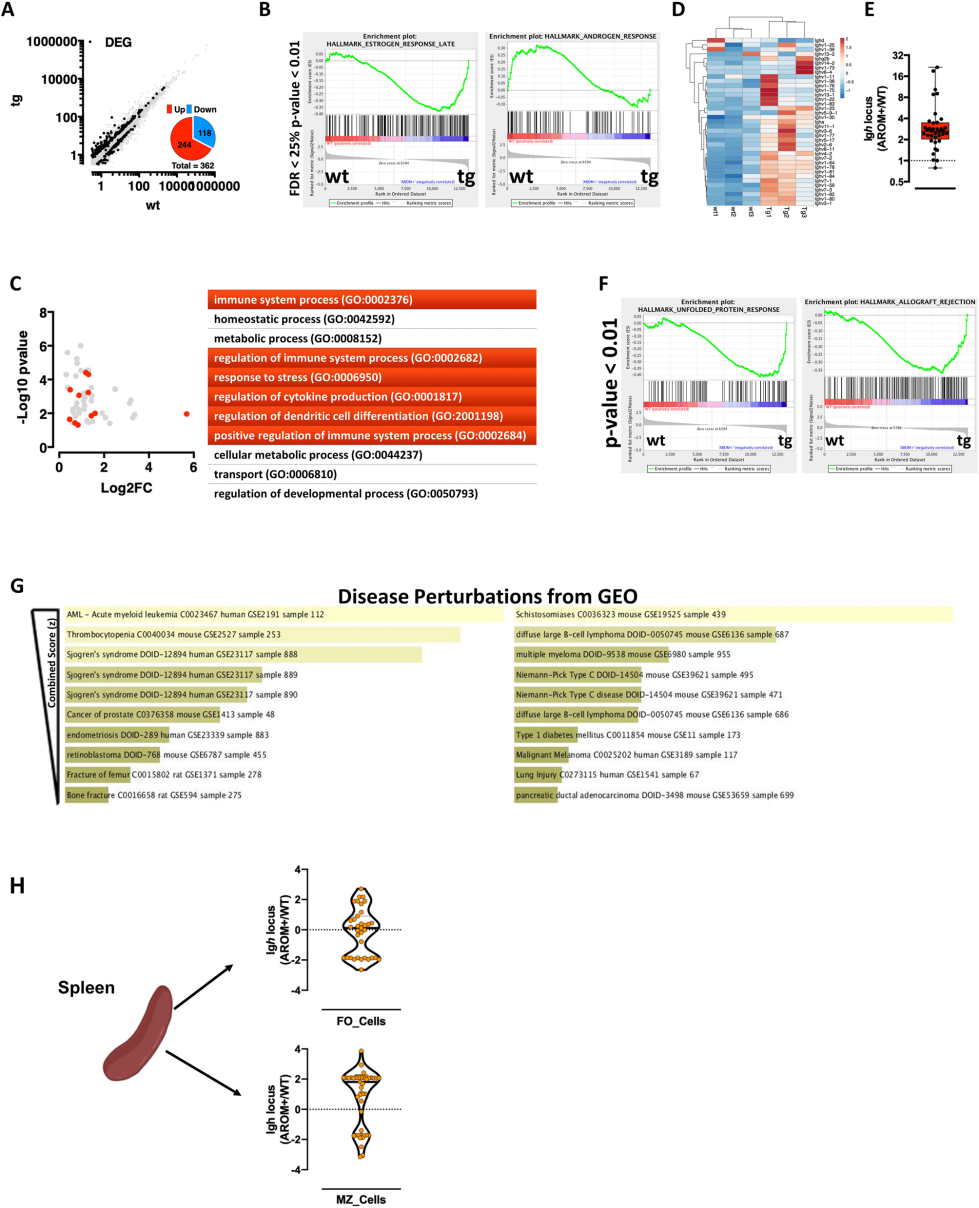
Transcriptome analysis of splenic B cells. (**A**) Scatter plot of splenic B cells transcriptome analysis. In black differentially expressed genes (DEGs), quantified in the pie chart. (**B**, **C**) Geneset Enrichment (**B**) and Gene Ontology Analysis (**C**) of DEGs. (**D**, **E**) Upregulation of the Igh locus shown as heatmap (**D**) and box plot (Log2FC AROM+/WT − E). (**F**) Geneset enrichment analysis highlighting over-representation of ER-Stress and autoimmunity genes in AROM+ B-Cells. (**G**) Enrichr-based disease perturbation analysis showing significant enrichment among DEGs of markers of human autoimmunity. (**H**) Violin plot (Log2FC AROM+/WT) of FO and MZ B-cell fractions showing the difference in *Igh* locus expression.

To study the molecular phenotype of the splenic B cells in AROM+ mice, we performed RNAseq profiling in isolated splenic total B cells from AROM+ and WT mice. The data showed a significant transcriptional perturbation of 362 transcripts, of which 244 are upregulated in AROM+ B cells (Fig. 8A) compared to B cells from the WT males. The GeneSet Enrichment Analysis (GSEA) revealed that the imbalance in E/A ratio results in altered B cell transcriptome, with significantly increased estrogen response and significantly decreased androgen responsive genes in AROM+ cells (Fig. 8B). This does not only indicate that B cells respond to circulating estrogen levels, but also suggests that most of the B cell responses in AROM+ males are dependent on the imbalanced E/A ratio. In keeping with the phenotypic analysis of the AROM+ animals, Gene Ontology enrichment of the differentially expressed genes between AROM+ and WT B cells highlighted an elevated immune reactivity of AROM+ B cells (Fig. 8C). This is in line with the strong and consistent upregulation of the Igh locus (Fig. 8D,E) and with the upregulation of genes involved in the unfolded protein response (generally activated in highly secretory cells) and systemic autoimmunity (Fig. 8F). The differentially expressed genes in AROM+ B cells as well as our in vivo and in vitro data described in the present study overlap with datasets of human autoimmune disorders, such as the Sjögren’s syndrome and Type 1 Diabetes (Fig. 8G). To map the transcriptional differences observed in splenic B cells to discrete subpopulations, we separated splenic B-cells into those homed in the follicular (FO) or the marginal zone (MZ) of the spleen and performed transcriptomic analysis. Interestingly, while functionally different, both B-cell subpopulations are significantly affected by the imbalanced E/A ratio (Supplementary Fig. 5). In keeping with their intrinsic different immune responsiveness, MZ B-cells from AROM+ males also display significant upregulation of the Igh locus (Fig. 8H).

All together, these data suggest that an imbalance in E/A ratio in males affects transcriptional responses in B-cells towards increased immune reactivity and individual susceptibility to autoimmune disorders.

## Discussion

Here we report that in the male AROM+ mice, that exhibit high circulating estrogen to androgen ratio, the levels of several immunoglobulin isotypes were increased starting at 60 days of age. Thus, the effect of E/A on the immunoglobulin production was evident after puberty. Longitudinal measures demonstrated that plasma immunoglobulin levels, particularly IgE, remained high in AROM+ mice over time. The role of sex steroids in the immunoglobulin production was confirmed by aromatase inhibitor (AI) treatment, which normalized the hormonal levels in AROM+ mice, and consequently decreased IgE concentrations. The notion that prolonged exposure to high E/A ratio leads to increased immunoglobulin secretion has been previously explained by the finding that estrogen receptor alfa (ESR1) activates the expression of genes coding activation-induced deaminase (AID), which in turn regulates both somatic hypermutation and class switch recombination^18^. In our RNAseq data on splenic B cells, the expression of AID genes was slightly but not significantly higher in AROM+ compared to WT males. However, we detected strong and consistent upregulation of the genes in the Igh locus. This is in accordance with a finding that the ESR1 binds directly to the regulatory elements in the Igh locus in vitro^19^, and thereby, has direct influence on antibody expression. Overall, our transcriptome analysis in splenic B cells showed a significantly increased expression of estrogen- and decreased expression of androgen-regulated genes, suggesting that B cells are a direct target of steroid hormones in AROM+ mice. Although, there are no previous studies showing the effect of estrogen and/or androgen on the global gene expression in the isolated splenic B cells, another study showed gender differences in the gene expression profile in the human peripheral B lymphocytes. In this study by Fan et al.^20^, 116 of 358 differently expressed genes in males and females were suggested to be estrogen regulated.

Above findings evidenced an altered humoral immunity in AROM+ mice, and therefore we studied the phenotype of splenic B cells. Exposure to increased E/T in AROM+ males modified the phenotype of splenic B cells as their proliferation rate and capability to produce IgE after immunoglobulin class switching in vitro was significantly increased compared to splenic B cells isolated from WT mice. Interestingly, the total amount of splenic and blood B lymphocytes was significantly lower, although the proportion of plasma cells, switched memory cells and double negative switched memory cells was higher in AROM+ compared to WT males. It has been shown earlier that estrogen treatment did not heighten the B220+ B cell population but instead foster immunoglobulin yield and cell growth of plasma cells^21^, which is in accordance with observation on AROM+ mice. Notably, the composition of B cell subsets in AROM+ males formed an individual cluster in the principal component analysis, indicating that estrogens regulate the splenic B cells in a sex-dependent manner. Furthermore, the dose-dependent effect of estrogen on immunoglobulin production by human PBMC may also envision the dissimilarity between AROM+ males and WT female mice^22^. Our observation is also supported by other work showing that estrogen promotes survival of autoreactive B cells by inhibiting apoptosis^4,23^, which might also explain the higher proportion of plasma and switched memory B cell populations, which we measured in the AROM+ mice. Furthermore, studies have indicated that estradiol treatment facilitates the high-affinity autoreactive mature B cell selection that occurs in the transitional 1 B cell to the transitional 2 B cell stage, and an elevated marginal zone B cells population^24,25^. The effect of estrogens on B cell maturation has been shown to occur via both estrogen receptors (ER), ERα and ERβ in female mice, while triggering autoimmunity seems to be solely ERα dependent^26^. With regard to androgens, they have previously found to regulate splenic B cells by suppressing their number both in male mice and men^27^. A more recent study also indicated, that androgens suppress the expression of BAFF cytokine in fibroblastic reticular cells (FRCs) in the spleen, and BAFF in turn, increases the number of the splenic B cells^28^.

Higher prevalence of allergic diseases and asthma has previously been linked to high estrogen concentrations or female gender in many studies^9,29–31^. The main contributors to above gender bias have been assumed to be sex steroids, such as estrogens, although other sex-specific factors such as sex chromosome dependent factors, sex-specific microbiota or behavioral aspects might also play a role. To test if AROM+ mice with high circulating E/T are more susceptible to allergic diseases than WT mice, we applied an allergic airway inflammation model. Our finding that the increase in IgE, and other immunoglobulin isotypes, was further enhanced in AROM+ males compared to WT males after challenge indicates that estrogens boost the immunoglobulin production. In previous studies, OVA challenge in female ovariectomized mice having low estrogen levels lead to decreased IgE levels^32^, and treatment of ovariectomized female mice with estrogenic compounds before ovalbumin immunization enhanced IgE production^5^. In line with this, we showed that female ArKO mice, having no circulating estrogens, had significantly lower plasma IgE levels than their WT littermates before and after OVA treatment. The production of basal levels of antibodies does not require hormonal stimulus, as we observed the same amount of IgE in plasma of ArKO and WT male mice before and after allergic challenge. Despite the strong effect on immunoglobulins, the hormonal imbalance in AROM+ male mice or ArKO female mice had no effect on the cellular infiltration or the composition of leukocytes in BAL after OVA challenge. This is in line with our observation that the Treg or T_FH_ cells were not altered in AROM+ compared to WT males. Interestingly, there is also another study showing that even high immunoglobulin levels do not always lead to pulmonary inflammation^33^.

Beyond the cellular analysis of the spleen, we observed that the spleen of AROM+ mice was significantly larger compared to WT animals. This was due to a diffuse follicular hyperplasia of the white pulp, while there was no evidence of an infection or neoplasia in the AROM+ mouse line. The exact origin and potential consequences of this enlargement are currently speculative and require further assessment. AROM+ mice have also been reported to have chronic inflammation in the testis and infertility^34^. We assume that the chronic inflammation in the testis of AROM+ mice do not have a role in the development of B cell-related phenotype, as the testicular inflammation develops during ageing, while changes in the humoral immunity are present already in young animals. Based on histology, there are no macrophage infiltration^35^, and the expression of Toll-like receptor 2 is not yet increased^36^, in AROM+ testis at the age of three months, while the changes in the antibody production can be measured already at the age of 48 days.

In addition to allergic diseases, sex steroids have been often linked to autoimmune diseases^37–39^. Accordingly, we found increased anti-DNA antibody concentration in plasma of the male AROM+ mice compared to WT males. This was in line with our global gene expression profile in splenic B cells isolated from AROM+ males, which resembled the data sets of human autoimmune diseases, such as Sjögren’s syndrome (SS) or type I diabetes. Interestingly, the incidence of SS is strongly female-biased, but almost all patients are postmenopausal women^31^. Female mice deficient in aromatase spontaneously develop SS resembling lesions^40,41^, characterizing the complex role of sex steroids in the development of autoimmune diseases.

Altogether, by coupling mouse genetics with state-of-the-art in vivo and molecular phenotyping, we show that an imbalanced E/T in male mice, at least partially, feminized immune response in the male mice. Our findings are in line with epidemiological data in humans showing an increased incidence of autoimmune disorders and allergies in females. The function of splenic B cells as well as their gene expression profile indicates that steroid hormones directly influence B cells in the spleen also in a sex-dependent manner. Defining the role of the genes in the activation of B cells leading to Ig production will not only help to understand the mechanism of class switching, but may also provide insights into defective IgE responses and allergic pathogenesis. Furthermore, considering the obvious sex-bias in many physiological and pathologic processes, such as in the severity of symptoms in recent COVID-19 infections^42^, our results highlight the importance of investigating both sexes differently also in animal studies aiming to drug discovery and therapeutic strategies.

## Materials and methods

### Mouse models

The in-house AROM+ (FVB/N) transgenic mouse model and the aromatase full knock-out ArKO (C576BL/6) mouse model, originally generated in the lab of Evan Simpson at the University of Texas Southwestern Medical Center, have been described previously^12,43^. The mice were given soy-free natural ingredient feed (Special Diets Services, Witham, UK) and tap water ad libitum, and housed in specific pathogen-free conditions at Central Animal Laboratory, University of Turku, complying with international guidelines on the care and use of laboratory animals. All animal handling was conducted in accordance with Finnish Animal Ethics Committee and the Institutional animal care policies of the University of Turku (Turku, Finland), which fully meet the requirements as defined in the NIH Guide on animal experimentation (NIH publication 86-23). For part of the studies, mice were shipped to the German Mouse Clinic (GMC). Mouse husbandry and all animal experiments were carried out in accordance with German legal guidelines and following the approval of the responsible animal welfare authorities and the ethics board of the district government of Upper Bavaria, Germany (full name: Regierung von Oberbayern)^44^.

### Immunoglobulin measurements in plasma

Blood was taken by a puncture of the saphenous vein, heart puncture, or retro-orbital plexus (Li-heparin-coated tubes; KADE, Nümbrecht, Germany) and centrifuged to separate cells and plasma. Total plasma IgE was measured in AROM+ and WT male mice at different age points using a classical immunoassay isotope-specific sandwich ELISA (BD Pharmingen). The determination of the immunoglobulin isotype levels (IgG1, IgG2a, IgG3, IgM and IgA) was performed either with a multiplex bead assay (Biorad, CA, USA) or with a combined electrochemiluminescence multiplexed assay system (Meso Scale Discovery, MSD, Rockville, MD USA).

### Measurement of anti-DNA antibodies

The levels of single- and double-stranded DNA autoantibody were determined with an indirect ELISA as previously described^45^. ELISA plates were coated with Poly-l-Lysine and calf thymus DNA (D4522-5 MG; Sigma Aldrich Chemie, Steinheim, Germany) for anti-DNA antibody detection. Plasma of 15 AROM+ and 15 WT male mice was diluted and loaded along with a serial dilution of a positive control [plasma of MRL/MpJ-Fas (lpr) mice]. Subsequently, goat anti-mouse secondary antibody (polyvalent IgG, IgA, and IgM; A0162-1ML, Sigma Aldrich Chemie) was added. After incubation, substrate was added and plates were read in a TECAN sunrise reader (Tecan Group, Maennedorf, Switzerland). In all immunoglobulin measurements, statistical significances were determined by the Mann–Whitney rank-sum test.

### Aromatase inhibitor treatment

To rescue the immunoglobulin levels of AROM+ male mice, 10 AROM+ and 10 WT mice were treated with aromatase inhibitor (AI, Finrozole CAS# 160146-17-8, Vetcare, Finland) and 10 AROM+ and 10 WT mice with vehicle only, for 6 weeks starting at the age of 4 weeks. AI (1 mg/ml) was given to mice daily with dose of 10 mg/kg of body weight by gavage. The vehicle was prepared as follows: 0.25 g carboxymethylcellulose (Sigma) was weighed and solubilized in 50 ml of deionized water. The solution was prepared once per week and stored at 4 °C. Aromatase inhibitor was weighed in a transparent glass mortar. A few drops of the vehicle were added, and the mixture was thoroughly mixed. Thereafter, one-third of the final volume of the vehicle was added to the mortar and placed into an ultrasonic incubator for 5 min. This procedure was completed three times to reach the final volume.

### Measurement of steroid hormone concentrations

Serum steroid hormone concentrations were measured in WT males, AROM+ males and in AROM+ males after aromatase inhibitor treatment. The concentration of estradiol-2, estradiol-1, T and A-dione were analyzed in a single run by validated gas chromatography tandem mass spectrometry method^46^. Briefly, after addition of isotope-labeled standards, steroids were extracted to cholorobutane, purified on a silica column, and derivatized using pentafluorobenzylhydroxyamine hydrochloride followed by pentafluorobenzoyl chloride. Steroids were detected with electron capture negative chemical ionization in multiple reactionmonitoringmode with ammonia as reagent gas using an Agilent 7000 triple guadrupole mass spectrometer. The lower limits of quantification were 0.5, 0.5, 8, and 12 pg/ml, respectively. The intraassay coefficients of variation were below 3.3% and the interassay coefficients of variation below 12.4%.

### Allergen-induced inflammation

Females or males 9–15 animals per group (AROM+ vs WT) or (ArKO vs WT) were sensitized and challenged to allergen as previously described^44,47^. In brief, mice were twice sensitized by i.p. injection of 10 µg OVA (Ovalbumin, Sigma, Germany) and 2 mg alum (inject-Alum Pierce, Rockford, USA) dissolved in 200 µl PBS at week 1 and week 2. Four weeks later mice were subsequently challenged three times by inhalative exposure to OVA aerosol (1% in PBS) for 30 min. One day after last aerosol challenge, animals were sacrificed, and blood collection and bronchoalveolar lavage (BAL) were performed.

### Measurement of cells in BAL fluid, blood and spleen

The degree of airway inflammation was studied by determination of differential cell counts on bronchoalveolar lavage fluid (BALF) by flow cytometric method as previously described^48^. BAL fluid cells were identified using the following monoclonal antibodies: anti-CD8a, anti-Ly6c, anti-CD4, anti-CD62L, anti-CD3, anti-CD25, anti-Gr1, anti-CD19, anti-CD11b, anti-CD11c, and anti-MHC-II^44^. Data were acquired using a LSRII flow cytometer (BD Bioscience) and further analyzed with FACSDiVa (BD Bioscience) and Flowjo V.7.2.2 (Tree star, Ashland, USA) software.

Spleen and blood cell staining follow the protocol that is applied in the phenotype screening at the GMC, and the flow cytometry gating strategy is shown in Supplementary Fig. 6. In brief, spleens were digested in enzyme buffer containing 10 mM HEPES, 5 mg/ml bovine serum albumin, 0.1 mg/ml DNAase I, and 0.6 mg/ml Collagease II at 37 °C for 30 min. The reaction was stopped by adding of EDTA in PBS. Subsequently, the spleens were homogenized by mechanical disruption via passing through a sterile 70 µm cell strainer. The isolated splenocytes were then subjected to RBC lysis buffer (eBioscience) to remove red blood cells. Thereafter, the blood cells and splenocytes were incubated with anti-CD16/32 (Fc-block; MR9-4, BD Biosciences) for 5 min and 15 min, respectively. Later, the FACS staining was performed at 4 °C with antibody mixture which contained anti-NK1.1-FITC (PK136, BD Biosciences), anti-CD21/CD35-PE (7G6, BD Biosciences), anti-I-A/I-E-PerCPCy5.5 (M5/114.15.2, BioLegend), anti-CD19-PE Cy7 (1D3, BD Biosciences), anti-Ly6G-APC (1A8, BD Biosciences), anti-CD5-APC (53-7.3, BD Biosciences), anti-CD45-Alexa Fluor 700 (30-F11, BioLegend), anti-CD11c-APCeFluor 780 (N418, eBioscience), and anti-CD11b-Pacific Blue (M1/70.15, Thermo Fischer Scientific) for 20 min, then followed by addition of Propidum iodide solution for live/dead cell discrimination. The lysis of erythrocytes from the blood samples was then performed by using BD Pharm Lyse (BD Biosciences). Additional staining for analysis of the B cell subsets was performed on splenocytes with above-mentioned procedure, but supplement of antibody mixture including anti-CD23-FITC (B3B4, BD Biosciences), anti-CD21/CD35-PE (7G6, BD Biosciences), anti-CD3e-PE CF594 (145-2C11, BD Biosciences), anti-CD38-PerCP eFlour 710 (90, eBioscience), anti-CD19-PE Cy7 (1D3, BD Biosciences), anti-IgM-APC (ll/41, eBioscience), anti-CD45-Alexa Fluor 700 (30-F11, BioLegend), anti-IgD-APC Cy7 (11-26c.2a, Biolegend), and anti-CD27-V450 (LG.3A10, BD Biosciences) and anti-CD138-BV510 (281-2, BD Biosciences). The gating strategy of B cell subset staining can be found at Supplementary Fig. 7. The cells collected from spleen cell proliferation assay also followed the procedure but with antibody mixture including anti-CD3e-PE CF594 (145-2C11, BD Biosciences), anti-CD19-PE Cy7 (1D3, BD Biosciences) and anti-CD45-Alexa Fluor 700 (30-F11, BioLegend). An introduction of the gating applied for the analysis of proliferated splenocytes was revealed at Supplementary Fig. 8. Data were acquired on Gallios Flow Cytometer (Beckman Coulter) and analyzed using FlowJo Software (Tree Star).

### Spleen cell proliferation and class switching in vitro

Single cell suspensions from spleen were prepared from 6 AROM+ and 6 WT males mice in 96-well plates with 200,000 cells per well. Assessment of Class Switch Recombination was done by stimulation with two concentration of a mixture of anti-CD40 antibody and IL4 (1 μg/ml anti-CD40+ 5 ng/ml IL-4 and 5 μg/ml anti-CD40+ 10 ng/ml IL-4) followed by a cultivation for 7 days. The CellTiter-Glo^®^ (Promega) Luminescent Cell Viability Assay kit was used for proliferation measurement following the manufacturer's instruction, and the luminescence was read on a Microplate Reader (TECAN Infinite M200).

### Histology

Histological analyses were performed in 32 mice, 12 AROM+ (6 females, 6 males) mice and 12 WT (6 females, 6 males) mice at the age of 19 weeks, and 5 AROM+ and 3 WT males at the age of 40 weeks. The tissues were collected, fixed in 4% neutral buffered formalin and embedded in paraffin. For histological examination, haematoxylin–eosin stain (HE) was routinely used. The immunohistochemistry (IHC) was performed using the streptavidin-peroxidase method with an automated immunostainer (DiscoveryXT; Roche, Penzberg, Germany) in paraffin-embedded tissue as reported previously^49^. After heat-induced antigen retrieval with citrate buffer, we used the following primary antibodies: anti-CD3 (SP7) from DCS Innovative Diagnostik-Systeme, Hamburg, Germany; anti-Cl597R06 and anti-CD45R/B220 from BD PharMingen, Heidelberg, Germany; 550286. Analyses were carried out in triplicates with an appropriate positive control. As negative control we stained the tissue without the primary antibody. Secondary antibody treatment and blocking against nonspecific binding were performed according to the information by the manufacturer. DAB was used for visualization of the antigen–antibody reaction. Membrane staining was considered as a positive reaction. Images for illustration were prepared by the slide-scanning system, NanoZoomer 2.0 HT (Hamamatsu Photonics K.K.; Hamamatsu City, Japan). The microscopic analysis was performed by experienced pathologists.

### RNA-Seq analysis

Splenic B cells were isolated from 3 AROM+ and 3 WT animals using the Miltenyi negative selection B-cell isolation kit (Miltenyi Biotec GmbH, Bergisch Gladbach, Germany). Total RNA was prepared with the Mini Elute RNA purification Kit (Qiagen) and 1 µg of purified RNA was sent to IGA Technology Services (https://www.igatechnology.com) for library preparation and mRNA-Seq. Briefly, libraries were prepared using the Ovation RNA-Seq System V2 (NUGEN) with Poly-A enrichment and sequenced at 75 bp-single ended using a HiSeq2500 Sequencing instrument (Illumina). About 40 million reads/sample were obtained. Classical bioinformatics analysis was performed following a Cufflink (default parameter)—Cuffdiff pipeline and differentially expressed genes were selected based on Fold Discovery Rate (FDR) < 25%. The list of differentially expressed genes was subsequently used for knowledge-based analysis methods such as Gene Ontology (https://pantherdb.org/tools/compareToRefList.jsp); GeneSet Enrichment Analysis (GSEA-Broad Institute); or combined pathway analysis (https://amp.pharm.mssm.edu/Enrichr/).

### Marginal Zone and Follicular B cells isolation

Spleen cells were enriched for MZ B cells (Marginal Zone CD21^hi^CD23^lo/–^) or FO B cells (Follicular CD21^int^ CD23^hi^) using a separation system (MACS; Miltenyi Biotech, Bergisch Gladbach, Germany). Total RNA was purified according Trizol^®^ protocol. RNA concentration was measured first through Qubit RNA BR Assay Kit (Thermo Fischer Scientific) and then ran on the Agilent Bioanalyzer chip to assess their integrity. Libraries were prepared using the QuantSeq 3’mRNA-Seq Library Prep Kit (cat # 015, Lexogen). At the second strand synthesis, UMI Second Strand synthesis Module were used in order to include identifiers in FWD libraries. Aliquots containing an equal number of nmoles of cDNA molecules from each library were pooled to obtain a pooled library with a concentration of 15 nM cDNA molecules. The pooled libraries were sequenced in an Illumina HiSeq2500 instrument (Illumina). Rapid Flow Cell 50 bp-SR (300 million reads). The reads were mapped to the mouse genome (nm10/GRCm38) by making use of the online platform “Bluebee”, as suggested from Lexogen (https://www.bluebee.com/lexogen).

### Statistical analyses

Statistical analyses were performed with GraphPad Prism 7 software (San Diego, CA, USA). Gaussian and non-Gaussian distributed results were further analyzed by unpaired t test or Mann Whitney test, respectively (GraphPad Prism, San Diego, CA, USA). Differences of P-values ≤ 0.05, ≤ 0.01, ≤ 0.001 and ≤ 0.0001 are shown as ns, *, **, *** and ****, respectively.

## Supplementary information

Supplementary Figures.

## References

[1] Roved J, Westerdahl H, Hasselquist D (2017). Sex differences in immune responses: Hormonal effects, antagonistic selection, and evolutionary consequences. Horm. Behav..

[2] Grossman CJ (1984). Regulation of the immune system by sex steroids. Endocr. Rev..

[3] Beagley KW, Gockel CM (2003). Regulation of innate and adaptive immunity by the female sex hormones oestradiol and progesterone. FEMS Immunol. Med. Microbiol..

[4] Altuwaijri S (2009). Susceptibility to autoimmunity and B cell resistance to apoptosis in mice lacking androgen receptor in B cells. Mol. Endocrinol..

[5] Sakai T (2010). The soy isoflavone equol enhances antigen-specific IgE production in ovalbumin-immunized BALB/c mice. J. Nutr. Sci. Vitaminol..

[6] Zaitsu M (2007). Estradiol activates mast cells via a non-genomic estrogen receptor-alpha and calcium influx. Mol. Immunol..

[7] Jensen-Jarolim E, Untersmayr E (2008). Gender-medicine aspects in allergology. Allergy.

[8] Straub RH (2007). The complex role of estrogens in inflammation. Endocr. Rev..

[9] Siroux V, Curt F, Oryszczyn MP, Maccario J, Kauffmann F (2004). Role of gender and hormone-related events on IgE, atopy, and eosinophils in the Epidemiological Study on the Genetics and Environment of Asthma, bronchial hyperresponsiveness and atopy. J. Allergy Clin. Immunol..

[10] Offner PJ, Moore EE, Biffl WL (1999). Male gender is a risk factor for major infections after surgery. Arch. Surg..

[11] Li X (2002). Mammary gland development in transgenic male mice expressing human P450 aromatase. Endocrinology.

[12] Li X (2001). Altered structure and function of reproductive organs in transgenic male mice overexpressing human aromatase. Endocrinology.

[13] Vehmas AP (2016). Liver lipid metabolism is altered by increased circulating estrogen to androgen ratio in male mouse. J. Proteom..

[14] Klein SL, Flanagan KL (2016). Sex differences in immune responses. Nat. Rev. Immunol..

[15] Abdullah M (2012). Gender effect on in vitro lymphocyte subset levels of healthy individuals. Cell Immunol..

[16] Griesbeck M (2015). Sex differences in plasmacytoid dendritic cell levels of IRF5 drive higher IFN-alpha production in women. J. Immunol..

[17] Lugli E (2007). Subject classification obtained by cluster analysis and principal component analysis applied to flow cytometric data. Cytometry Part A J. Int. Soc. Analyt. Cytol..

[18] Pauklin S, Sernandez IV, Bachmann G, Ramiro AR, Petersen-Mahrt SK (2009). Estrogen directly activates AID transcription and function. J. Exp. Med..

[19] Jones BG (2016). Binding of estrogen receptors to switch sites and regulatory elements in the immunoglobulin heavy chain locus of activated B cells suggests a direct influence of estrogen on antibody expression. Mol. Immunol..

[20] Fan H (2014). Gender differences of B cell signature in healthy subjects underlie disparities in incidence and course of SLE related to estrogen. J. Immunol. Res..

[21] Verthelyi DI, Ahmed SA (1998). Estrogen increases the number of plasma cells and enhances their autoantibody production in nonautoimmune C57BL/6 mice. Cell Immunol..

[22] Kanda N, Tamaki K (1999). Estrogen enhances immunoglobulin production by human PBMCs. J. Allergy Clin. Immunol..

[23] Grimaldi CM, Cleary J, Dagtas AS, Moussai D, Diamond B (2002). Estrogen alters thresholds for B cell apoptosis and activation. J. Clin. Investig..

[24] Grimaldi CM, Michael DJ, Diamond B (2001). Cutting edge: expansion and activation of a population of autoreactive marginal zone B cells in a model of estrogen-induced lupus. J. Immunol..

[25] Grimaldi CM, Jeganathan V, Diamond B (2006). Hormonal regulation of B cell development: 17 beta-estradiol impairs negative selection of high-affinity DNA-reactive B cells at more than one developmental checkpoint. J. Immunol..

[26] Hill L, Jeganathan V, Chinnasamy P, Grimaldi C, Diamond B (2011). Differential roles of estrogen receptors alpha and beta in control of B-cell maturation and selection. Mol. Med..

[27] Sakiani S, Olsen NJ, Kovacs WJ (2013). Gonadal steroids and humoral immunity. Nat. Rev. Endocrinol..

[28] Wilhelmson AS (2018). Testosterone is an endogenous regulator of BAFF and splenic B cell number. Nat. Commun..

[29] Rubtsova K, Marrack P, Rubtsov AV (2015). Sexual dimorphism in autoimmunity. J. Clin. Investig..

[30] Takeda M (2013). Gender difference in allergic airway remodelling and immunoglobulin production in mouse model of asthma. Respirology.

[31] Konttinen YT (2012). Sex steroids in Sjogren's syndrome. J. Autoimmun..

[32] Riffo-Vasquez Y, de Oliveira APL, Page CP, Spina D, Tavares-de-Lima W (2007). Role of sex hormones in allergic inflammation in mice. Clin. Exp. Allergy J. Br. Soc. Allergy Clin. Immunol..

[33] Mathias CB (2009). IgE influences the number and function of mature mast cells, but not progenitor recruitment in allergic pulmonary inflammation. J. Immunol..

[34] Li X (2006). Transgenic mice expressing p450 aromatase as a model for male infertility associated with chronic inflammation in the testis. Endocrinology.

[35] Strauss L (2009). Increased exposure to estrogens disturbs maturation, steroidogenesis, and cholesterol homeostasis via estrogen receptor alpha in adult mouse Leydig cells. Endocrinology.

[36] Mayer C (2016). Sterile inflammation as a factor in human male infertility: Involvement of Toll like receptor 2, biglycan and peritubular cells. Sci. Rep..

[37] Kanda N, Tsuchida T, Tamaki K (1999). Estrogen enhancement of anti-double-stranded DNA antibody and immunoglobulin G production in peripheral blood mononuclear cells from patients with systemic lupus erythematosus. Arthritis Rheum..

[38] Tabor DE, Gould KA (2017). Estrogen receptor alpha promotes lupus in (NZBxNZW)F1 mice in a B cell intrinsic manner. Clin. Immunol..

[39] Tessnow AH, Olsen NJ, Kovacs WJ (2011). Expression of humoral autoimmunity is related to androgen receptor CAG repeat length in men with systemic lupus erythematosus. J. Clin. Immunol..

[40] Shim GJ (2004). Aromatase-deficient mice spontaneously develop a lymphoproliferative autoimmune disease resembling Sjogren's syndrome. Proc. Natl. Acad. Sci. USA.

[41] Iwasa A (2015). Aromatase controls Sjogren syndrome-like lesions through monocyte chemotactic protein-1 in target organ and adipose tissue-associated macrophages. Am. J. Pathol..

[42] Gebhard C, Regitz-Zagrosek V, Neuhauser HK, Morgan R, Klein SL (2020). Impact of sex and gender on COVID-19 outcomes in Europe. Biol. Sex Differ..

[43] Fisher CR, Graves KH, Parlow AF, Simpson ER (1998). Characterization of mice deficient in aromatase (ArKO) because of targeted disruption of the cyp19 gene. Proc. Natl. Acad. Sci. USA.

[44] Fuchs H (2011). Mouse phenotyping. Methods.

[45] Angelis MHD, Chambon P, Brown S (2006). Standards of Mouse Model Phenotyping.

[46] Nilsson ME (2015). Measurement of a comprehensive sex steroid profile in rodent serum by high-sensitive gas chromatography-tandem mass spectrometry. Endocrinology.

[47] Aguilar-Pimentel JA (2010). Specific CD8 T cells in IgE-mediated allergy correlate with allergen dose and allergic phenotype. Am. J. Respir. Crit. Care Med..

[48] Daubeuf F (2017). A fast, easy, and customizable eight-color flow cytometric method for analysis of the cellular content of bronchoalveolar lavage fluid in the mouse. Curr. Protoc. Mouse Biol..

[49] Kunder S (2007). A comprehensive antibody panel for immunohistochemical analysis of formalin-fixed, paraffin-embedded hematopoietic neoplasms of mice: Analysis of mouse specific and human antibodies cross-reactive with murine tissue. Toxicol. Pathol..

