# Supplementary material for: Increased estrogen to androgen ratio enhances immunoglobulin levels and impairs B cell function in male mice
Source: Sci Rep. 2020 Oct 27;10:18334. doi: 10.1038/s41598-020-75059-9 (PMC7591566; doi:10.1038/s41598-020-75059-9)

# Increased estrogen to androgen ratio enhances immunoglobulin levels and impairs B cell function in male mice

Juan Antonio Aguilar-Pimentel **^1,*^**, Cho Yi-Li **^1^**, Raffaele Gerlini **^2,3^**, Julia Calzada-Wack **^1^**, Maria Wimmer **^4,5^**, Philipp Mayer-Kuckuk **^1^**, Thure Adler **^6,7^**, Carsten B. Schmidt-Weber ^5^, Dirk H. Busch **^6^**, Helmut Fuchs **^1^**, Valérie Gailus-Durner **^1^**, Markus Ollert **^8,9^**, Martin Hrabě de Angelis **^1,2,3,10,*^**, Claes Ohlsson**^11^**, Matti Poutanen **^11, 12, 13^**, Raffaele Teperino **^2,3^,** Leena Strauss **^12,13^**

**Supplement Figure 1. Sex-bias in the amounts of natural killer (NK) cells and eosinophils in blood.** The figure shows the relative amounts of natural killer (NK) cells (A) and eosinophils (B) in the blood samples. The AROM+ males resembled more to the WT females than to the WT males (median, n= 10).


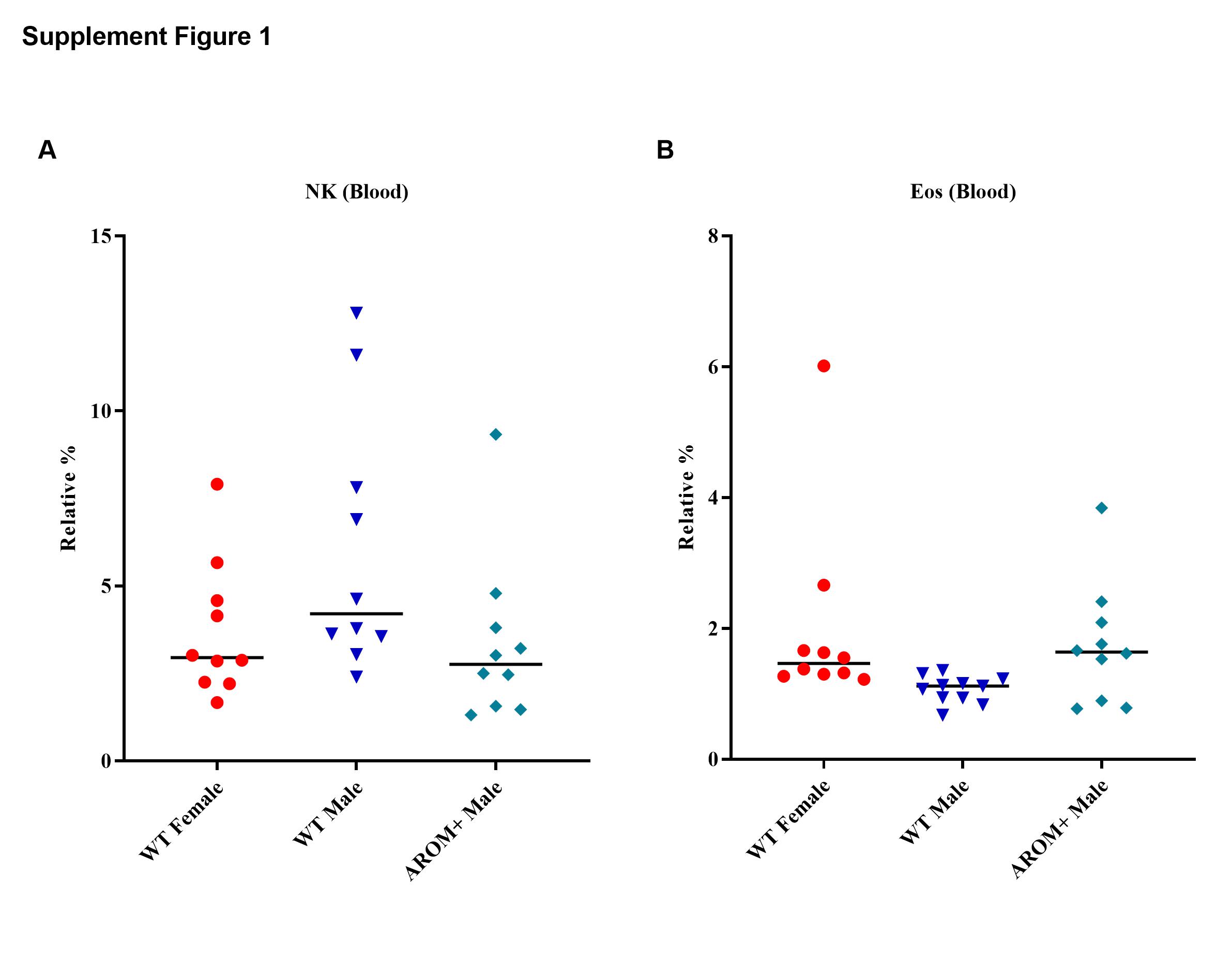


**Supplement Figure 2. Sexual dimorphism in the immune cells.** A) Relative distribution of T and B cells in the spleen of male and female mice as determined by a large-scale phenotyping screening from mice at the German Mouse Clinic (GMC, data of C57BL6 WT mouse screened). B) Correlation of the spleen weight and body weight showing heavy spleens in females when correlated to males. Median, n> 340 , Mice between 15 to 17 weeks old (C57BL/6).


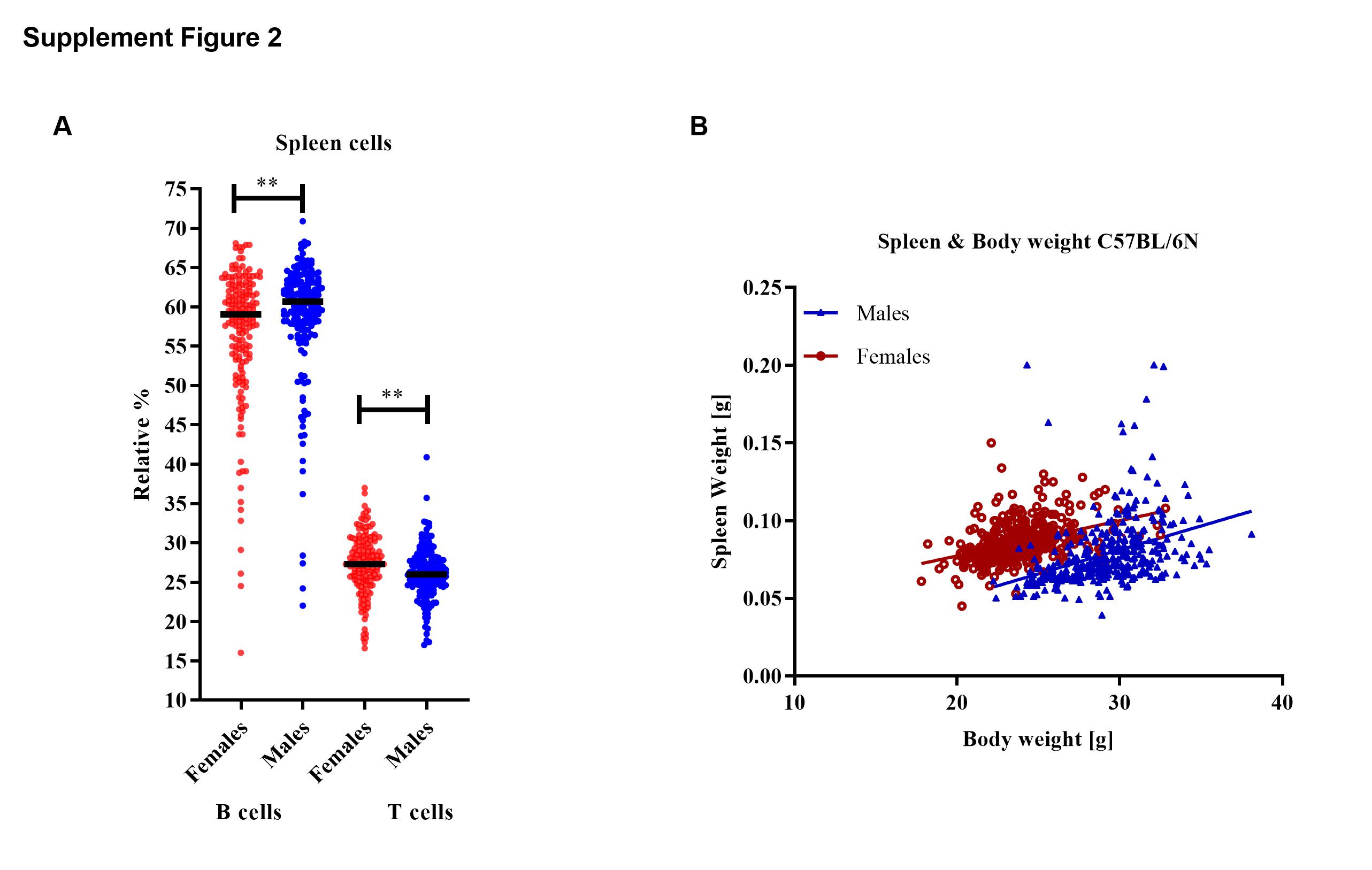


**Supplement Figure 3. Immunohistochemical detection of B and T cells in the spleen.** The B and T cells were detected by immunohistochemical staining for B220 and CD3, respectively. Representative micrograph of longitudinal sections of the spleen at low magnification is presented. Using immunohistochemistry (IHC) a subtle enlargement of the follicular area in the AROM+ mutant mice was detected, without any significant difference between the proportion of T and B lymphocytes (n= 3-5).


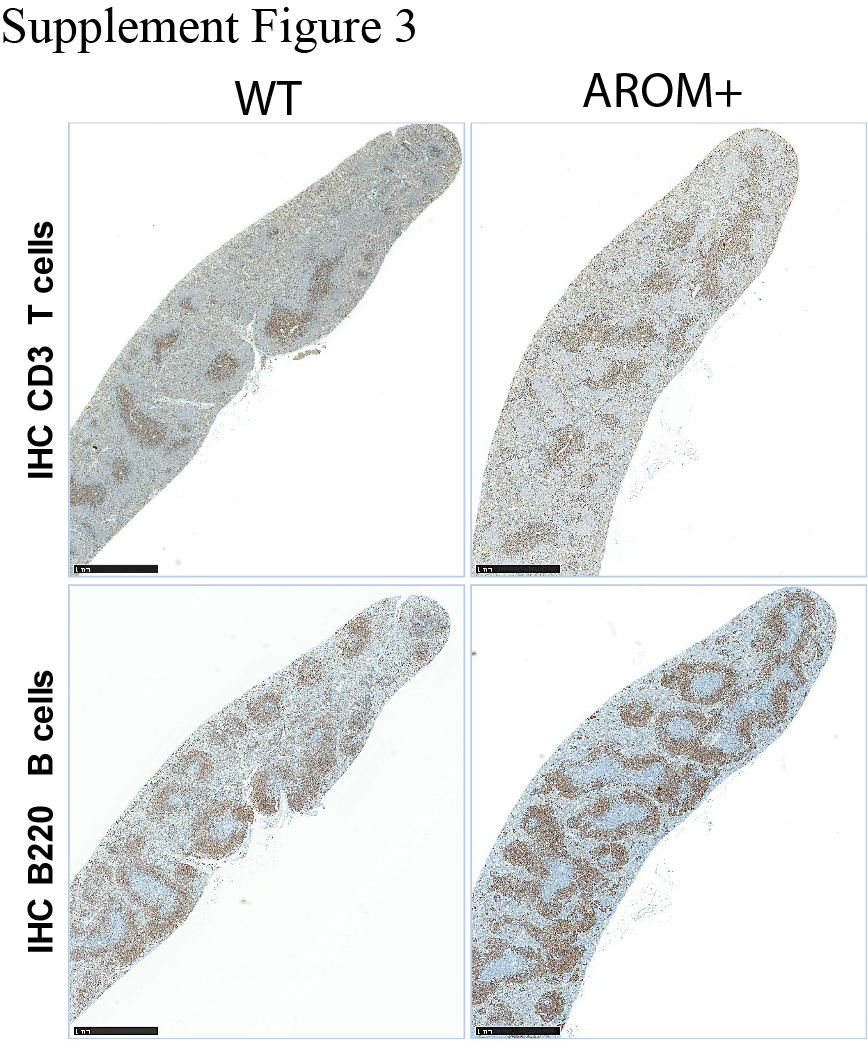


**Supplement Figure 4. Flow cytometric immunophenotyping of the small intestine, Peyer’s patches and spleen.** Cells were isolated from the small intestine (SI), Peyer’s patches (PP) and spleen. Cells were further labelled with monoclonal antibodies specific for CD45, CD3, CD4, RORgt, Foxp3, GATA3 and Helios for the identification of T Cells (Treg), or labelled with CD45, CD4, PD1, CXCR5, Icos, and Foxp3 for the identification of follicular helper T cells (TFH). Overall, no differences were detected in the percentage of regulatory Treg or TFH or subsets between the spleens of AROM+ and WT mice. Data is presented as mean ±SD).


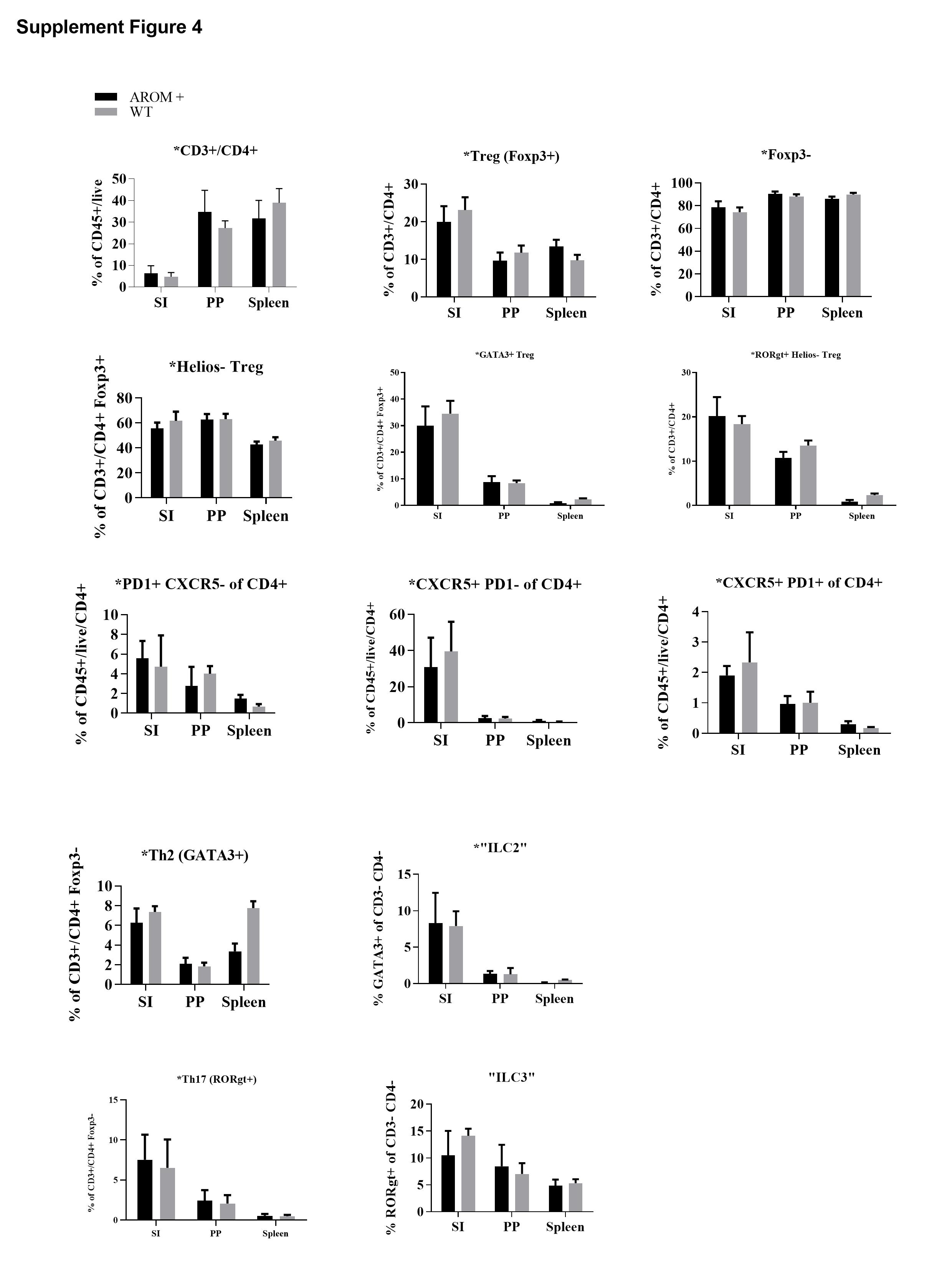


**Supplementary Figure 5. Bar plots of the GO analysis of Molecular Function.** A-B) Scatter plot of normalized read counts per gene (average of three replicates) calculated by EdgeR in WT and AROM*+* MZ (left A) and FO (left B) cells. The black dots represent the genes called differentially expressed with p-value <0.05 and fold change >0.5 and <-0.5. The numbers of genes upregulated and downregulated are indicated in the cake plots. The bar plot shows Fold Enrichment values of the significant enrichment terms of genes involving their molecular function in FO (right A) and MZ (right B) isolated cells.


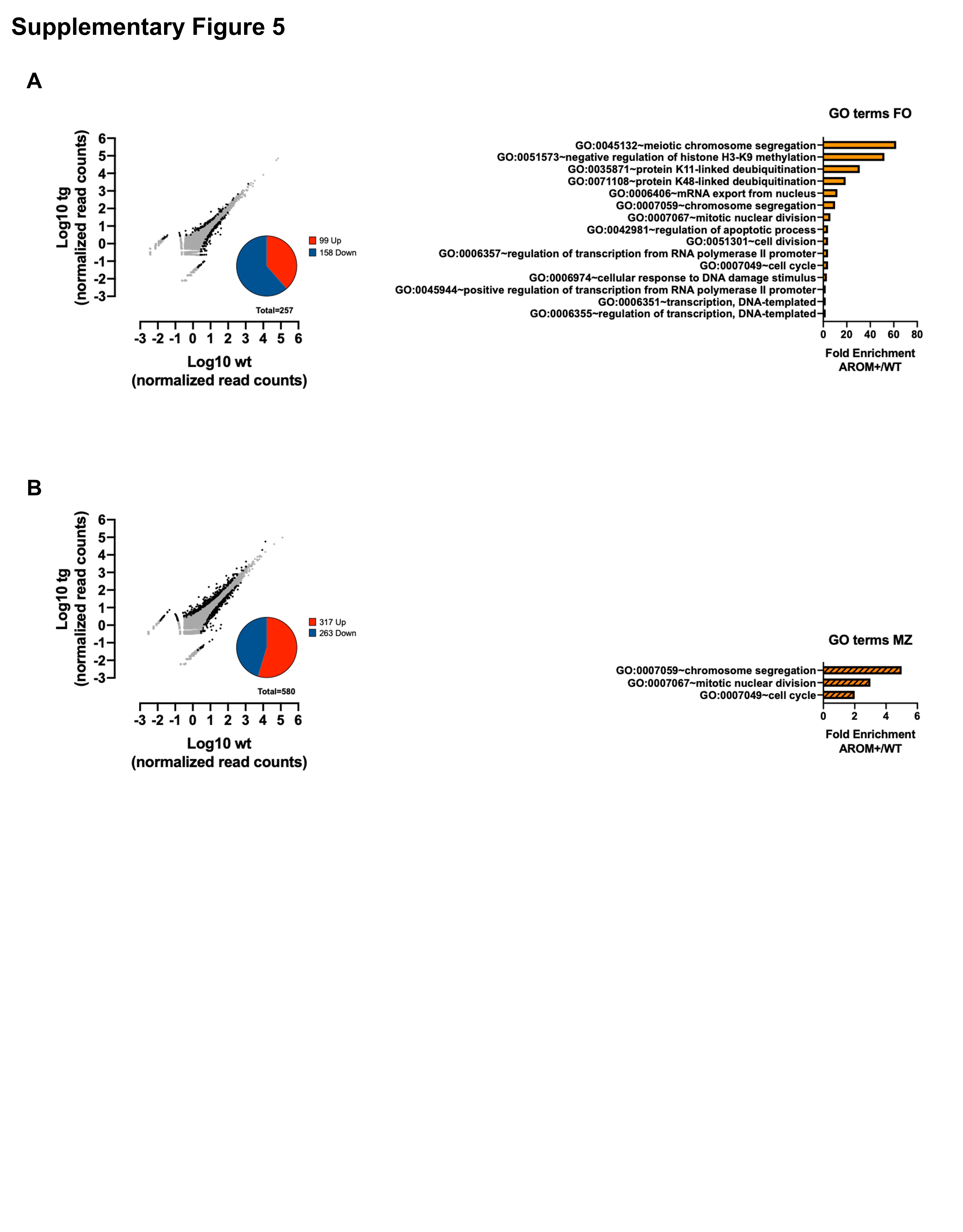


**Supplement Figure 6. Flow cytometry gating strategy.** Example of broad immune phenotyping gating strategy by flow cytometry using FlowJo**.** Spleen cells were first gated on the singlet cells (FSC-A vs. FSC-W), singlet cells were then analyzed for the expression of leukocyte common antigen CD45, and the dead cells were excluded by PI staining, then a gate was applied to include the cells of interest


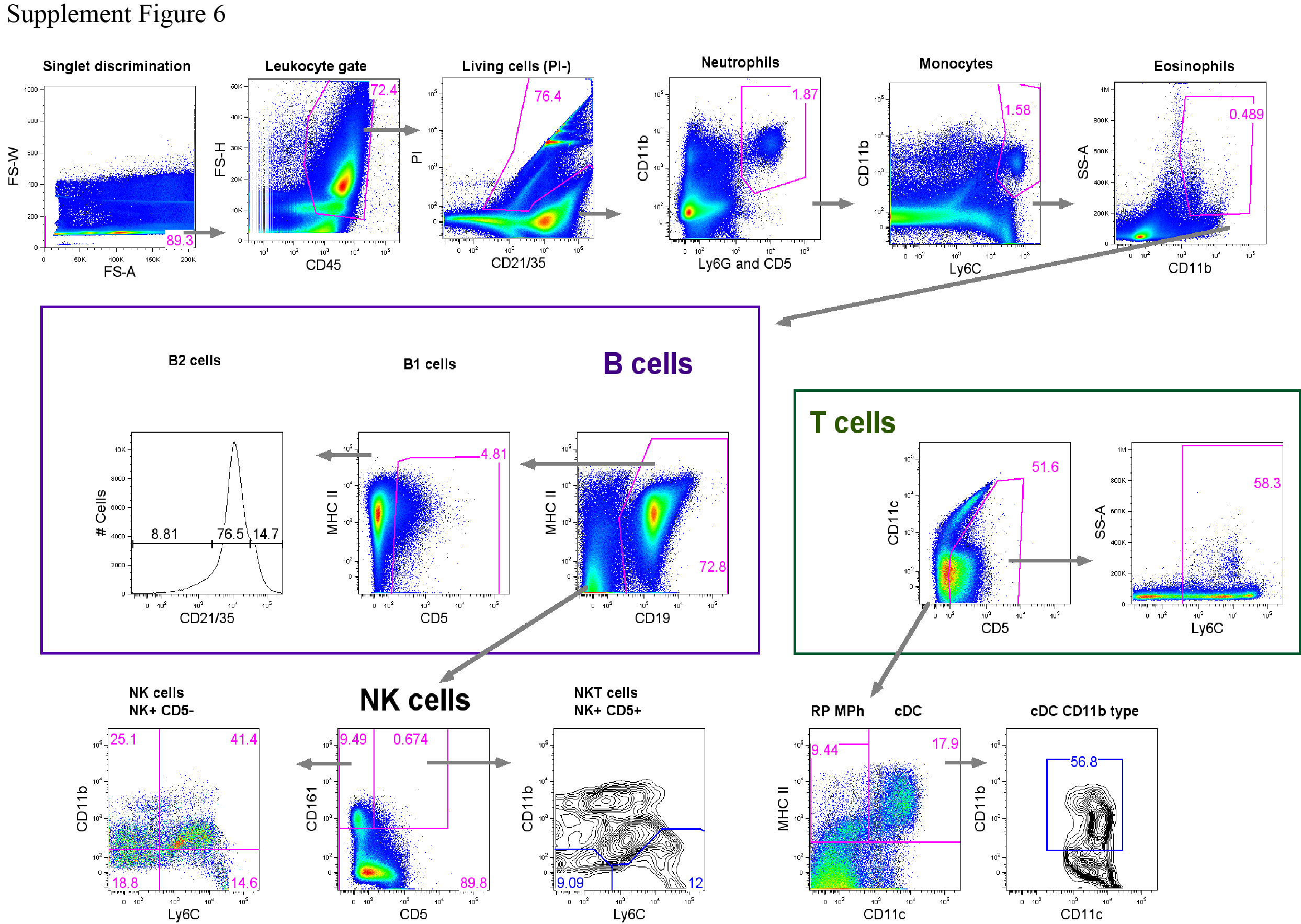


**Supplement Figure 7. Exemplary analysis of multi-color FACS staining for identification of the specific B cell subsets.** Spleen cells were first gated for lymphocytes (SSC-A vs. FSC-H) and singlet cells (FSC-H vs. FSC-W). The singlet cells were then analyzed for the expression of leukocyte common antigen, CD45, and the dead cells were excluded by PI staining. The live cells were further divided by the expression of CD3 and CD19 markers, defining the T cells as CD3+ cells and B cells as CD19+ cells. Thereafter, the detailed B cell subsets were distinguished from the gated CD19+ B cell population and indicated in the figure.


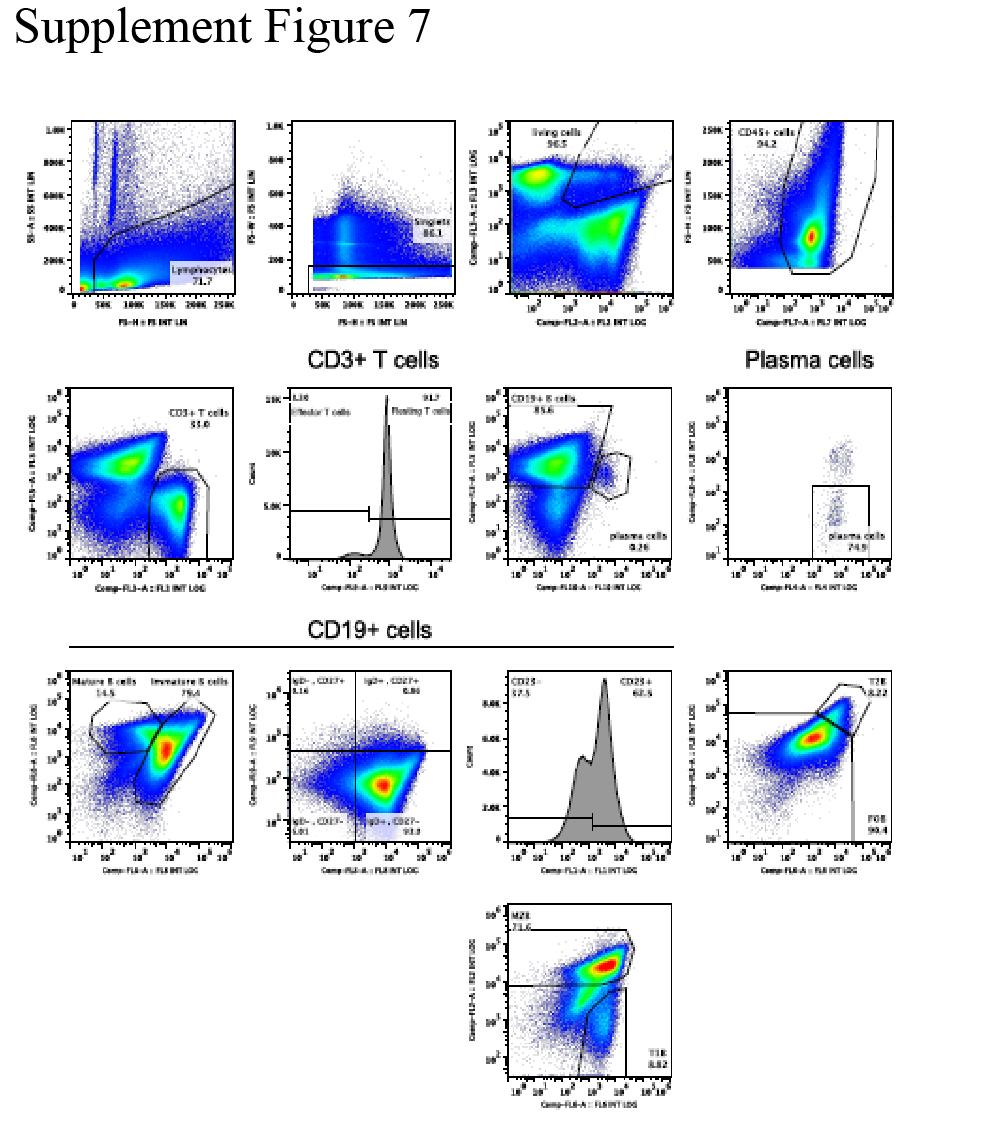


**Supplement Figure 8.** **Representative plot of FACS staining analysis at day-7 after cell stimulation *in vitro*.** Stimulated splenocytes were first gated for lymphocytes (SSC-H vs. FSC-H) and singlet cells (FSC-H vs. FSC-A). The singlet cells were then analyzed for the expression of leukocyte common antigen CD45 and excluded the dead cells by PI staining. The T cells were then defined as CD3+ cell population and B cells as CD19+ cell population from gated live CD45+ leukocytes.


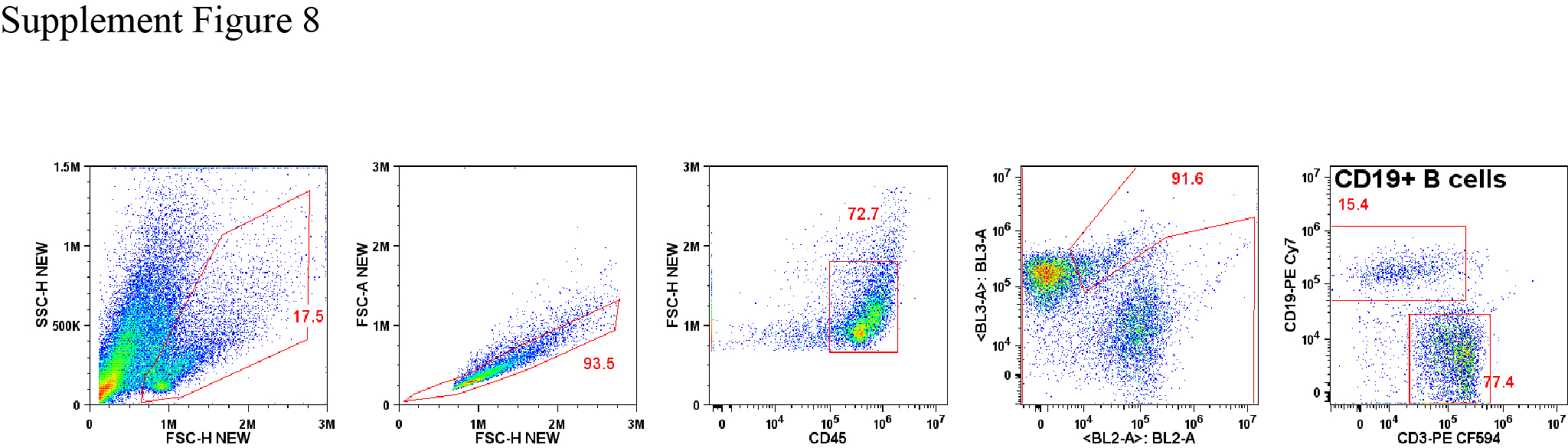

Supplement: Supplementary file 1 — Supplementary Figures. [file 41598_2020_75059_MOESM1_ESM.docx]
